# Comparing the intra-tumoral distribution of Gemcitabine, 5-Fluorouracil, and Capecitabine in a murine model of pancreatic ductal adenocarcinoma

**DOI:** 10.1371/journal.pone.0231745

**Published:** 2020-04-16

**Authors:** Louise M. Fanchon, James Russell, Nagavarakishore Pillarsetty, Isabella O’Donoghue, Kishore Gangangari, Kenneth H. Yu, John L. Humm

**Affiliations:** 1 Department of Medical Physics, Memorial Sloan Kettering Cancer Center, New York, NY, United States of America; 2 Department of Radiology, Memorial Sloan Kettering Cancer Center, New York, NY, United States of America; 3 Department of Chemistry, Hunter College, City University of New York, New York, NY, United States of America; 4 Gastrointestinal Oncology Service, Memorial Sloan Kettering Cancer Center, New York, NY, United States of America; Vrije Universiteit Brussel, BELGIUM

## Abstract

**Purpose:**

To develop a technique to compare the intra-tumoral distribution of the drug gemcitabine, its surrogate [^18^F]-fluoroarabinocytosine ([^18^F]-FAC) and related chemotherapeutics 5-FU and capecitabine in a pre-clinical model of pancreatic ductal adenocarcinoma (PDAC).

**Experimental design:**

Using a KPC-organoid derived model of PDAC, we obtained autoradiographic images of the tumor distribution of, [^14^C]-gemcitabine, [^14^C]-5-FU, [^3^H]-capecitabine. These were compared indirectly by co-administering [^18^F]-FAC, a close analog of gemcitabine with a proven equivalent intra-tumor distribution. The short half-life of ^18^F allows for clean separation of ^3^H/^14^C labeled drugs in specimens by dual isotope digital autoradiography. Autoradiographic images of [^14^C]-gemcitabine, [^3^H]-capecitabine and [^14^C]-5-FU were each correlated to [^18^F]-FAC on a pixel-by-pixel basis. The tumor drug penetration was compared using cumulative histograms.

**Results:**

Gemcitabine distribution correlated strongly with FAC as expected. 5-FU also gave a similar microdistribution to that of FAC, whereas no correlation was found between capecitabine or its metabolic products and FAC distribution. Accumulation of Gemcitabine and 5-FU was lower in hypoxic regions of the tumor, whereas no such correlation was observed for capecitabine and its metabolites.

**Conclusions:**

Gemcitabine and 5-FU target the same regions of the tumor, leaving hypoxic cells untreated. Capecitabine metabolites penetrate further into the tumor but it is yet to be determined whether these metabolites are the active form of the drug.

## Introduction

Pancreatic ductal adenocarcinoma (PDAC) remains one of the deadliest types of cancer with a five-year survival rate around 5% [1]. In nearly 80% of cases, pancreatic cancer is diagnosed too late to be operable and no cure is currently available for those patients [2, 3]. Gemcitabine monotherapy has been the standard of care for more than a decade before getting replaced by a two or three-drug regiment [4], gemcitabine combined with nab-paclitaxel and the FOLFIRINOX regime (5-fluorouracil (5-FU), leucovorin, irinotecan and oxaliplatin) being the current standard of care [5, 6]. Recently two phase 3 trials found that combining gemcitabine and capecitabine significantly improved survival for patients with resected pancreatic ductal adenocarcinoma [7] and for patients with unresectable locally advanced or metastatic disease [8]. Neoptolemos *et al*. advanced the hypothesis that gemcitabine and capecitabine act synergistically. An alternative explanation is that the patient population contained individuals whose tumors responded to one agent or the other [9]. In this scenario, individual patients would not benefit greatly from receiving two agents per se, though they would be more likely to receive one effective agent.

The poor response of pancreatic cancer to chemotherapy is ascribed to both poor vascularization and the large amount of tumor stroma that generates high interstitial tumor pressure and thus limits drug penetration into the tissue [10]. This barrier is successfully recapitulated in genetically engineered KPC mice that express both mutant K-ras and TP53 in pancreatic tissue, leading to spontaneous PDAC formation. In these models it has been shown that doxorubicin and gemcitabine do not penetrate far into the tumor [11], and it seems likely that most chemotherapy agents would be similarly restricted to perivascular regions. Multiple studies and clinical trials have been performed to assess drug toxicities and test drug combinations, but no study has compared intra-tumoral drug distribution.

In this study we determined the spatial distribution of gemcitabine, 5-FU and metabolized capecitabine in an organoid mouse model of pancreatic cancer. The organoid culture was established from a tumor growing spontaneously in a KPC mouse and used to generate a transplantable tumor model. It is worth noting that KPC tumors have been shown to be similarly responsive to gemcitabine and capecitabine and that the combination GEMCAP was found to have an additive effect [12].

We determined the spatial distributions through autoradiography using ^14^C labeled gemcitabine and 5-FU and ^3^H labeled capecitabine. Capecitabine is a prodrug for 5-FU, that undergoes a three-stage activation with the final conversion step occurring inside the tumor [13, 14]. 5-FU is further metabolized, yielding both therapeutic and non-therapeutic end products. Thus, the signal on the autoradiographs represents a mixture of chemical species. The main inactive 5-FU product is fluoro β alanine (FBAL). The ^14^C label of 5-FU is on the C2 position, which is lost from FBAL. However, for capecitabine the tritium label is retained on all metabolites. In consequence, the autoradiographic distribution of capecitabine is not necessarily an image of active compound (S1 Fig). To compare the distributions of 5-FU and capecitabine metabolites with gemcitabine in the same tumor we used an ^18^F labeled gemcitabine analog and potential PET tracer [^18^F]-FAC [15]. Because ^18^F has a 110-minute half-life, it is possible to co-administer it with long -lived ^3^H (or ^14^C) labeled compounds and separate the isotopic contribution of each. FAC is identical to gemcitabine, except that it is mono- rather than difluorinated at the 2 position. The radiolabeling of FAC to [^18^F]-FAC is much easier than labeled gemcitabine with ^18^F [15]. Our prior studies have shown that the uptake and spatial distribution of [^18^F]-FAC spatially correlates well with gemcitabine in another mouse model of pancreatic cancer [16]. By comparing the spatial distributions of [^3^H]-capecitabine and [^18^F]-FAC, we can infer the extent to which gemcitabine and capecitabine metabolites share a common destination within the tumor.

## Materials and methods

### Animals and tumor model

All experiments were carried out with the knowledge and approval of the Institutional Animal Care and Use Committee (IACUC) of Memorial Sloan Kettering Cancer Center (MSKCC). Similarly, the animals used in this study were cared for in accordance with guidelines approved by MSKCC Institutional Animal Care and Use Committee and Research Animal Resource Center. The MSK vivarium, where the animals were housed, is an IACUC and AAALAC approved facility. A total of 15 C57Bl/6 mice (Jackson Laboratories, Bar Harbor ME) were used in this study; Tumor implantation was performed by trained staff. Animals were placed under general anesthetic (isoflurane inhalation) coupled with 2 mg/kg of meloxicam /0.5 mg/kg buprenorphine subcutaneously as pre-emptive analgesia. Tumor fragments (approximately 2 mm) were transplanted by stitching to the exteriorized pancreas with ligature (4–0 Vicryl, polyglactin 910, Ethicon, Bridgewater, NJ). On completion of surgery, the muscle layer was closed using Vicryl, skin edges were closed with sterilized wound clips and a local anesthetic (bupivacaine) was applied at the incision edges. Post-op care included close monitoring of the animals and administration of meloxicam once/day for 2 days as analgesia. Wound clips were removed one week after surgery. Animals were housed five per cage with food (PicoLab Rodent Diet 5053) and water ad libitum. Cages are checked twice daily and changed weekly. Mice were on a 12/12 light cycle. The animals tolerated the procedure well and euthanasia (by CO_2_ inhalation at a prescribed flow rate of 3 liters/minute) was only required to terminate the experiment. CO_2_ inhalation is a humane and rapid method of euthanasia that matches the recommendations of the Panel on Euthanasia of the American Veterinary Medical Association.

Organoid cultures derived from a KPC mouse were the generous gift of Dr David A. Tuveson (Cold Spring Harbor, NY). Organoids were cultured using serum-free advanced DMEM/F12, supplemented with B27 (1:50) and Noggin (100 ng/ml), all from GIBCO, (Waltham MA); and EGF (25 ng/ml); FGF-10 (100 ng/ml); (Leu15)-Gastrin-1 (210 ng/ml); N-Acetyl Cysteine (1 μmole/ml); Nicotinamide (10 μmole/ml) Y-27632 (10 nmoles/ml), all from Sigma Aldrich, (St Louis MO) and R-spondin1 medium (1:10). R-spondin 1 medium was collected from 293T-HA-Rspo1-Fc (Sigma) cells, grown to confluence, and then maintained in that state in serum-free advanced DMEM/F12 for 7 days. Organoids were cultured in 48 well plates in Matrigel, overlaid with growth medium. After expansion, the contents of the inner 24 wells were pooled and injected orthotopically in to the pancreas of a single a C57BL/6 mouse.

The resulting tumor was chopped into approximately 2 mm fragments and grafted onto the pancreas, for further serial passage [17]. Established tumors were found to be mycoplasma free by reculture in antibiotic free medium (MycoAlert, LonzaPlus, Basel, Switzerland). Tumors used in this study were at the third passage. Growth of the tumors was monitored using ultrasound imaging, until they reached a final size of approximately 1 cm. The average time to reach that size was 3 weeks (ranging from 2.5 weeks to 1 month).

### Radioisotopes

[^14^C]-gemcitabine, [^14^C]-5-FU and [^3^H]-capecitabine, were obtained from Moravek Biochemicals (LaBrea, CA). Lot number 154-110-055-A-20131114-AAH, category MC2246 for [^14^C]-gemcitabine, lot number 945-119-0576-A-20160510-PVA, category MC101 for [^14^C]-5FU and lot number 580-097-001-A-20100603-TN, category MT1874 for [^3^H]-capecitabine. Gemcitabine and 5-FU were labeled on the 2 position of the pyrimidine ring; capecitabine on the 6 position. [^18^F]-FAC (2'-deoxy-2'-[^18^F]fluoro-β-D-arabinofuranosylcytosine was synthesized in-house as previously described [15].

### Autoradiography and image analysis

All animals received [^18^F]-FAC (250 μCi/animal) and pimonidazole (100 mg/kg, NPI, Burlington MA) injected intraperitoneally. Animals also received either [^14^C]-gemcitabine (2 μCi), ^14^C-5FU (2 μCi), or ^3^H-capecitabine (25 μCi). Radioisotope was administered in a treatment dose of unlabeled drug: 40 mg/kg gemcitabine 20mg/kg 5-FU, or 250 mg/kg capecitabine (all drugs from Sigma Chemicals, St Louis, MO) Gemcitabine and 5-FU were given intraperitoneally, co-administered with [^18^F]-FAC and pimonidazole; capecitabine was dissolved in 5% gum Arabic in 40 mM citrate buffer, pH 6, and given by oral gavage. Two hours after injection the mice were sacrificed, the tumor was extracted and frozen on dry ice in Tissue-Tek Optimal Cutting Temperature compound (OCT, Sakura, CA) and immediately sectioned. 10-μm thick sections were cut at the widest cross section. Sections were exposed on autoradiography plates for 2 hours at -20° C and scanned on a Typhoon scanner to obtain an image of the [^18^F]-FAC distribution. 48 hours later, after complete decay of the ^18^F activity, autoradiography of the second tracer (radiolabeled with ^14^C or ^3^H) was initiated. Sections were exposed at– 80^o^ C for two weeks to obtain an image of either ^14^C-gemcitabine distribution or ^14^C-5-FU or for 3 months for ^3^H-capecitabine. BAS-IP MS 2025 (Fujifilm, Japan) imaging plates were used for ^14^C and ^18^F and BAS-IP TR 2025 (Fujifilm, Japan) imaging plates were used for ^3^H. Imaging plates were scanned on a Typhoon FLA 7000 laser scanner (GE Healthcare Life Sciences, Pittsburgh, PA, USA).

^18^F and either ^14^C or ^3^H images were obtained from the same section. Prior to autoradiography, fiduciary markers containing ^18^F and ^14^C in nail varnish (20 and 0.2 nCi/μl respectively) had been placed on the slides [18] to register the two autoradiographs. Necrotic areas identified by H&E staining were masked out of the autoradiographs for analysis. The registered and masked image pairs were analyzed in ImageJ (National Institutes of Health, Bethesda, MD), to generate 10–50,000 pairs of values, each pair representing the ^18^F and ^14^C/^3^H intensity at one location in the section. The correlation between ^18^F and ^14^C or ^3^H intensity was obtained in Microsoft Office ProPlus Excel. Pixel values were also used to generate cumulative histograms showing the distribution of activity in the tumor. To measure the gemcitabine and [^18^F]-FAC colocalization the correlation coefficient was calculated. The two autoradiography images were registered together with the H&E image, and a mask was drawn manually on the [^18^F]-FAC autoradiography image to delineate the tumor region, excluding the necrotic regions as seen on the H&E image. The mask was then applied to the gemcitabine autoradiography. The pixel coordinates for each image inside the mask was recorded and the correlation coefficient calculated. The same process was applied to capecitabine images. Because the capecitabine and 5-FU images were weak (due to the low energy of the tritium decay and the rapid metabolism of 5-FU in the liver [12], we enhanced the signal-to noise ratio in ImageJ by registering 3–5 adjacent sections and generating a median image (S2 Fig). The resulting image was then correlated to the [^18^F]-FAC image as described above.

### Immunohistochemistry

Sections exposed for autoradiography were stained with Hematoxylin and Eosin (H&E) while adjacent sections were used for immunohistochemistry, as follows. Sections were fixed in ice-cold methanol for 30 minutes. Staining was done with biotinylated hyaluronic acid binding protein (HABP, EMD Millipore Corp, MA) and antibodies against collagen (Abcam, Cambridge MA, cat# ab34710), pimonidazole (Hypoxyprobe, Burlington, MA) and Meca-32, (The Developmental Studies Hybridoma Bank, Iowa City IA). For hyaluronic acid staining, sections were blocked with an Avidin-Biotin blocking kit as per manufacturer’s instructions (Vector Labs, Burlingame CA), 0.3% H_2_O_2_ (15 minutes); and 1% bovine serum albumin (BSA), for 1 hour at room temperature. Sections were exposed to HABP in 1% BSA overnight at 4°C, and subsequently washed three times in PBS. Signal was developed by ABC reagent followed by diaminobenzidine (both Vector Labs) as per manufacturer’s instructions For antibody staining, sections were blocked in 1% bovine serum albumin; primary antibody was applied for 1 hour followed by washing and secondary (anti-rabbit alexafluor 488 or anti-rat alexafluor 568 (Life Sciences Scientific, NJ)) for 1 hour. Sections were imaged with an Olympus BX60 microscope and Microsuite Biological Suite imaging software (Olympus America, Center Valley PA, USA). The images obtained from autoradiography were registered with the stained sections (H&E, Meca-32 and Pimonidazole) using Adobe Photoshop (Adobe, San Jose, CA). To establish tumor hypoxic fractions, pimonidazole images were thresholded in ImageJ, using the Otsu automated segmentation procedure, which creates two subpopulations with the minimum possible total variation.

### Scintillation counting and gamma-counting

Prior to processing for cryosectioning, approximately 100mg of tumor was cut off and weighed. ^18^F was counted in the Wallac Wizard 1840 Gamma Counter (Perkin Elmer, Waltham, MA), after which tissue was solubilized (Solvable^TM^, Perkin Elmer, MA) and ^14^C- or ^3^H- beta decays were counted on the Tricarb 2910 TR liquid scintillation counter (Perkin Elmer, MA).

### Statistics

Descriptive statistics used in the text are the mean ± the standard deviation (S.D.), and the correlation coefficient, all calculated in Excel. Correlations were established between autoradiograph images on a pixel-by-pixel basis, where images contained up to 10^5^ pixels. Because of this, the statistical significance of the correlation coefficients (r) is not informative, as even very low values of r yield p < 0.05.

## Results

### Animals and tumor model

**Fig 1:**
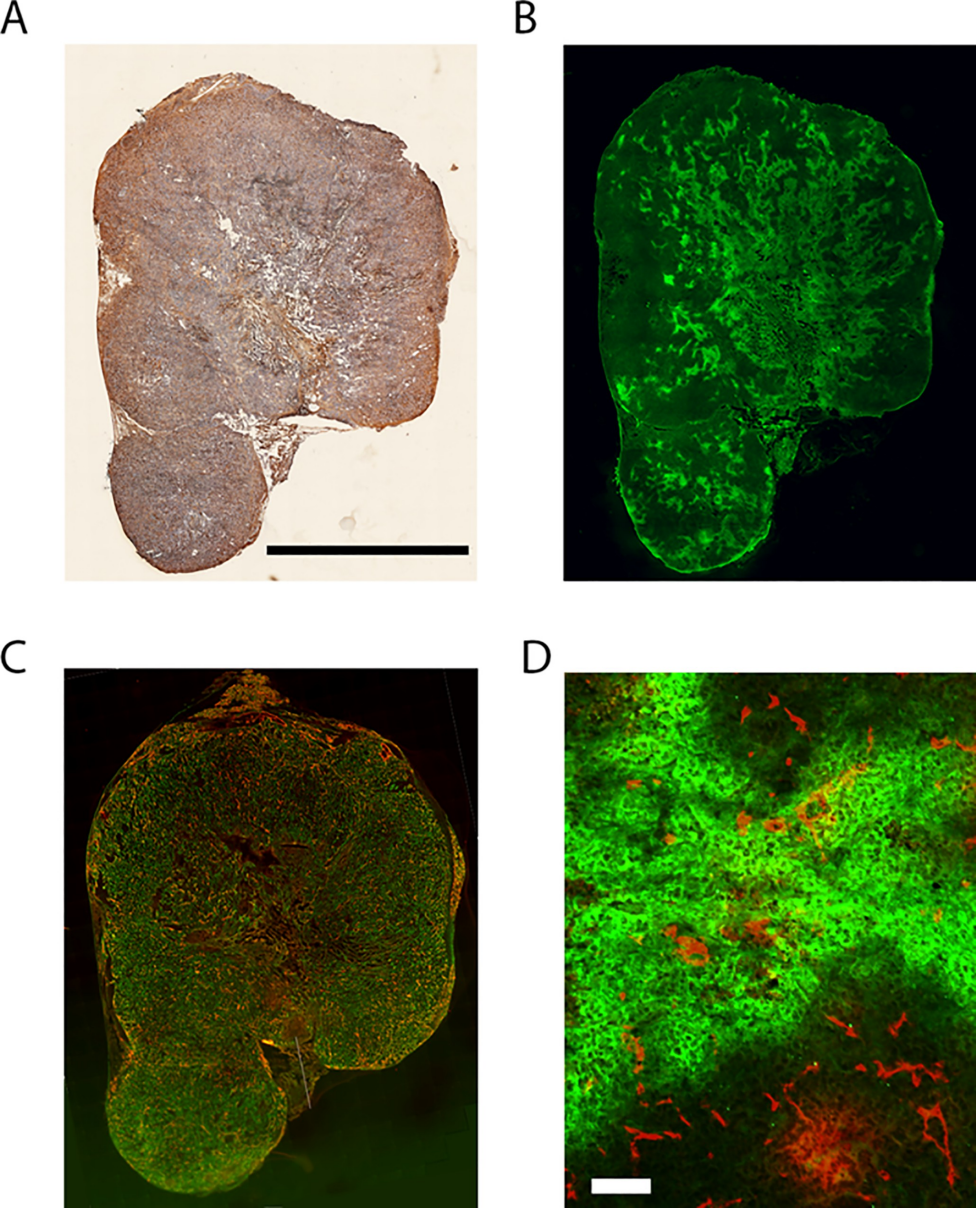
KPC-derived organoid tumor histopathology features. KPC-derived organoid tumor stained for hyaluronic acid (A), pimonidazole (B), collagen (green) and Meca32 (red) (C). Co-staining of pimonidazole (green) and Meca-32 (red) (D) showing vessels present in both oxic and hypoxic tissue. Black scale bar = 5 mm. White scale bar = 100 μm.

To verify that KPC-derived organoid tumors used in this study exhibited the same characteristics as pancreatic tumors, tumor sections were stained for hyaluronic acid (Fig 1A), pimonidazole (Fig 1B), collagen and Meca32 (Fig 1C). The representative section shown in Fig 1 shows abundant hyaluronic acid particularly in the tumor periphery and significant levels of collagen. Fig 1C shows that the vascularization is interweaved in with bands of collagen, the latter constituting a barrier to drug penetration. Automatic segmentation of the pimonidazole-stained sections (from five tumors, one section per tumor) gave the number of hypoxic pixels to the number of oxic pixels ratio to be 0.47 with a standard deviation of the mean of 0.16. Although the tumors were extremely hypoxic, they were relatively well vascularized as shown in Fig 1D where vessels are present in both oxygenated and hypoxic tissue suggesting that not all vessels were perfused.

**Fig 2:**
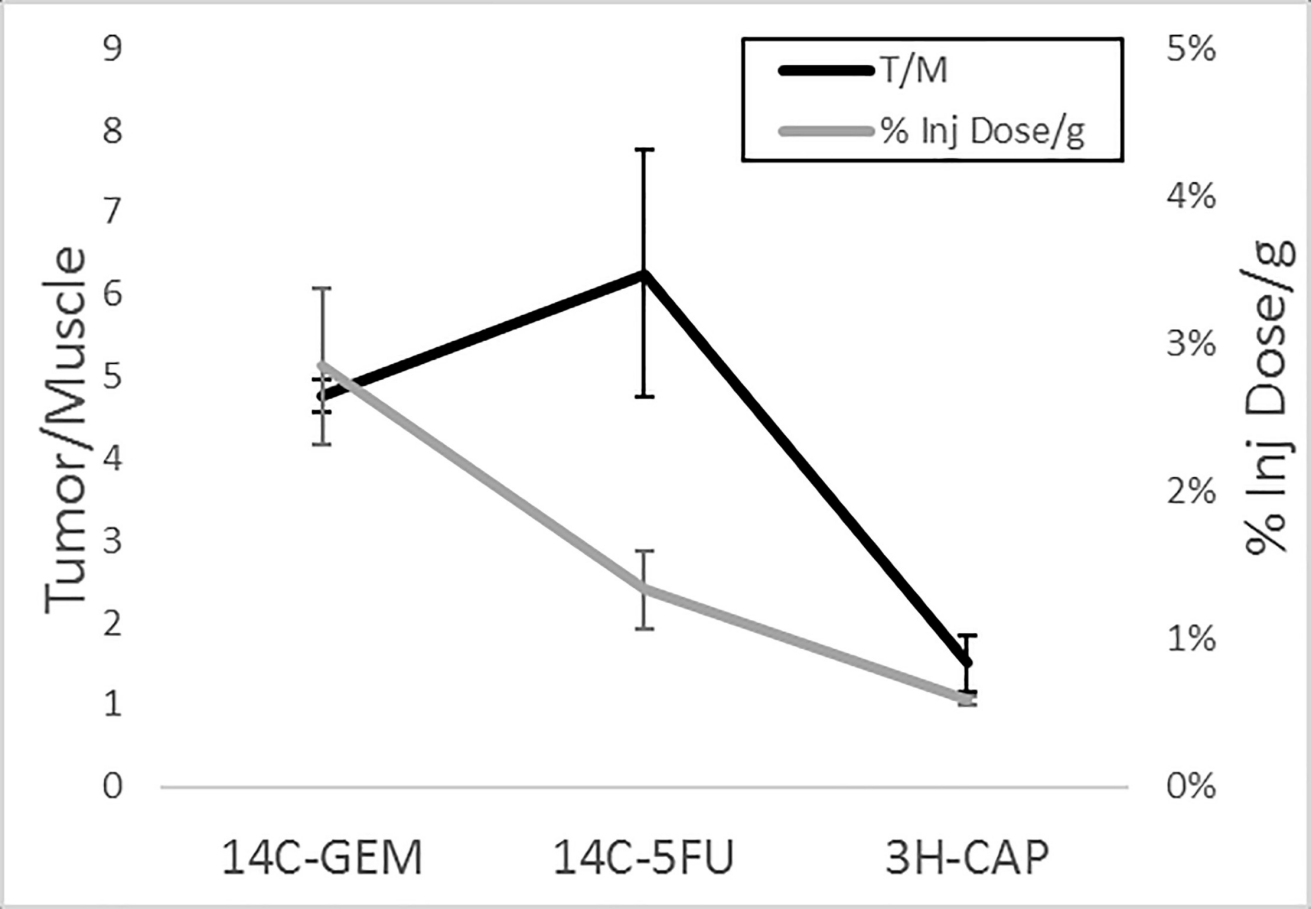
Gemcitabine, capecitabine and 5-FU tumor uptake. Tumor-to-muscle ratio (black line) and percentage injected dose per gram (gray line) of [^14^C]-Gemcitabine, [^14^C]-5-FU and [^3^H]-Capecitabine, measured by scintillation counting of the radiolabeled drug in tissue samples taken from 5 mice per tracer. Mean ± standard deviation.

We determined gross uptake of gemcitabine 5-FU and capecitabine as measured from scintillation counting for 5 mice per group at two hours post injection. To calculate the percent injected dose per gram. (Fig 2), the amount of injected activity was obtained from an aliquot of the injected solution. As an indicator of tumor specific uptake, results were further standardized to the percent of dose in muscle expressed as the tumor-to-muscle (T/M) ratio (defined as the activity per gram of tumor, relative to that of muscle). The T/M is of the same order for 5-FU and gemcitabine (4.8 ± 0.2 (S.D.) and 6.2 ± 1.5 (S.D.) respectively) whereas it is lower for capecitabine with a mean ratio of 1.5. However, capecitabine is a pro-drug so activity in the muscle may represent the inactive form. The percentage injected dose per gram is lower for 5-FU and capecitabine than for gemcitabine.

### Validation of [^18^F]-FAC colocalization with [^14^C]-gemcitabine

We studied the co-localization of the drugs by comparing the spatial distribution of each agent to [^18^F]-FAC, a close analog of gemcitabine. The autoradiographic images are the product of beta particles of different mean energies (6 keV for ^3^H, 49 keV for ^14^C, and 250 keV for ^18^F) where low energy betas give a weaker signal, but with a higher resolution. Because of the rapid decay of ^18^F, images of ^3^H and ^14^C (collected 48 hours or 26 ^18^F half-lives later) are free of any ^18^F signal. The imaging plates used for ^18^F image capture are completely insensitive to ^3^H decay. However, the initial 2-hour exposure used to collect the ^18^F images will have some contribution from ^14^C. This problem can be circumvented by administering excess of the ^18^F construct, so that the signal contribution of the ^14^C to the first image plate read-out is reduced to insignificance. This was verified by performing a 2-hour autoradiographic plate exposure upon full decay of the ^18^F, to assess the magnitude of the ^3^H/^14^C contribution to the ^18^F composite image. It was shown that the ^14^C activity contribution to the first plate read-out was less than 1%. To obtain ^14^C images, autoradiographic plates were exposed for 2–3 weeks prior to read-out. ^3^H decays can be completely masked by covering the section with 10 μm plastic wrap, to eliminate the contribution of ^3^H decays from the ^18^F autoradiograph.

**Fig 3:**
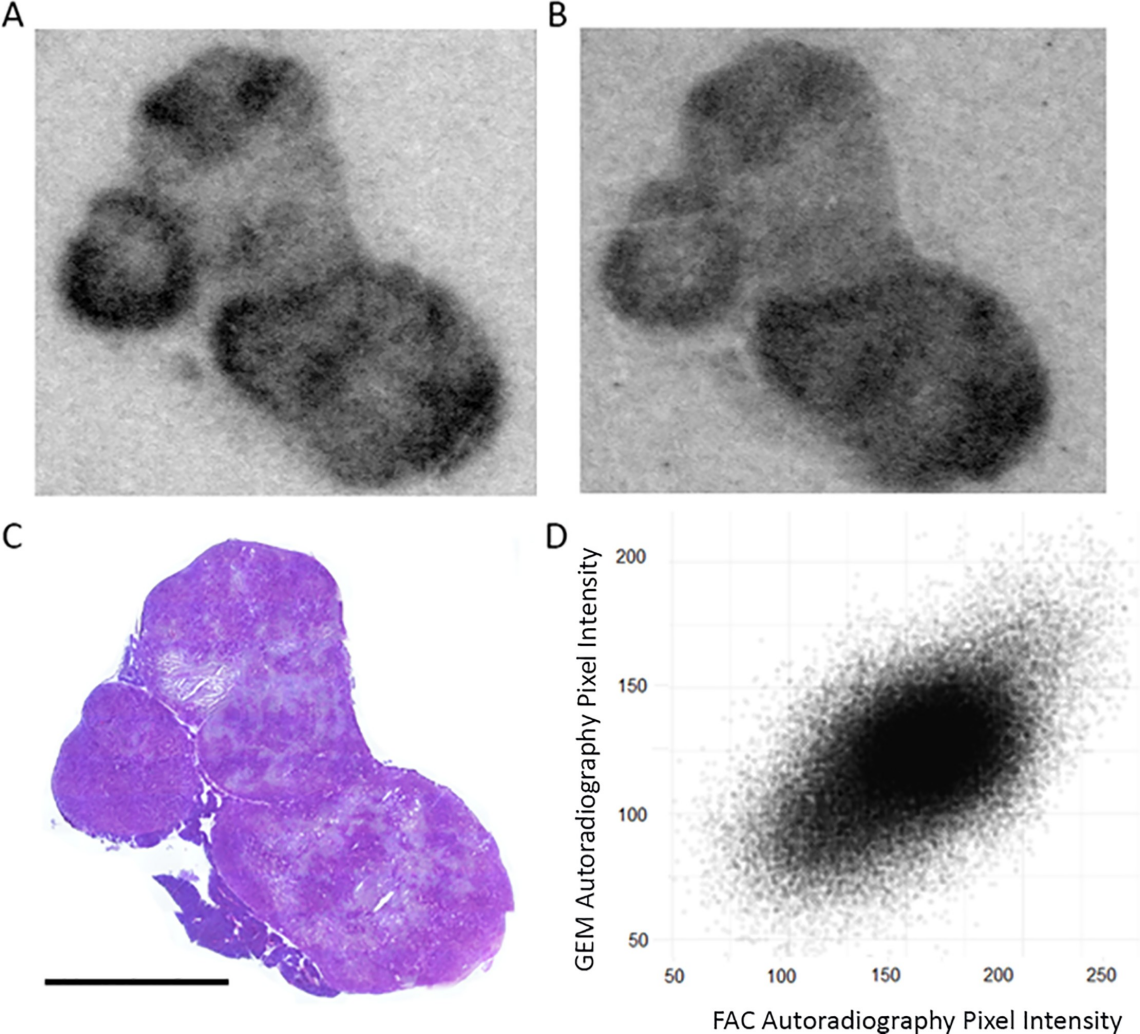
Gemcitabine and FAC co-localization. Autoradiography of [^14^C]-gemcitabine (A) and of [^18^F]-FAC (B) in an organoid tumor section. H&E staining of that tumor section (C) and pixel to pixel correlation of pixel intensity between [^14^C]-Gemcitabine and [^18^F]-FAC autoradiography (D). Scale bar is 5 mm.

In Fig 3, we show concordance between the distributions of [^14^C]-gemcitabine and [^18^F]-FAC in a tumor section, with the complete data set (n = 4) provided in S3 Fig. For both isotopes, although necrotic tissue, identifiable in the corresponding H&E images is clearly “cold”, the viable tissue also contains regions of both low and high activity accumulation. By registering the ^18^F and ^14^C images, we were able to correlate pixel values for the non-necrotic tissue; a sample scatter plot is shown in Fig 3D. For the complete data set, the average correlation coefficient between [^14^C]-gemcitabine and [^18^F]-FAC was 0.65, implying that 42% (r^2^) of the variation in [^14^C]-gemcitabine can be explained in terms of [^18^F]-FAC. This is likely an underestimate, since there are potential errors which can affect the correlation, particularly small image mis-registrations, and differences arising from the physical differences in the point spread function of the two radionuclides. The difference in the range of the emitted beta particles will result in difference in image resolution and the difference in half-life will affect the image noise, reducing the agreement between [^18^F]-FAC and [^14^C]-gemcitabine. The good agreement between gemcitabine and FAC allowed us to use [^18^F]-FAC as a surrogate for [^14^C]-gemcitabine for the subsequent experiments mapping the distribution of 5-FU and capecitabine.

### Capecitabine metabolites and 5-FU distribution compared to that of [^18^F]-FAC

We sought to understand the intra-tumoral distribution of 5-FU and capecitabine. We co-administered [^18^F]-FAC with either radio-labelled capecitabine or 5-FU, allowing us to indirectly compare the distribution of these agents to gemcitabine. As capecitabine is a prodrug that is metabolized to 5-fluorouracil, which itself undergoes further metabolism, the autoradiograph will not represent a single chemical entity. The same concerns apply to the 5-FU images; however, the position of the ^14^C label (on C2 of 5-FU) means that the main inactive metabolite, FBAL, will be unlabeled (S1 Fig). In the following discussion we refer to “capecitabine-derived” and “5-FU derived” images to indicate that several chemical species are involved.

**Fig 4:**
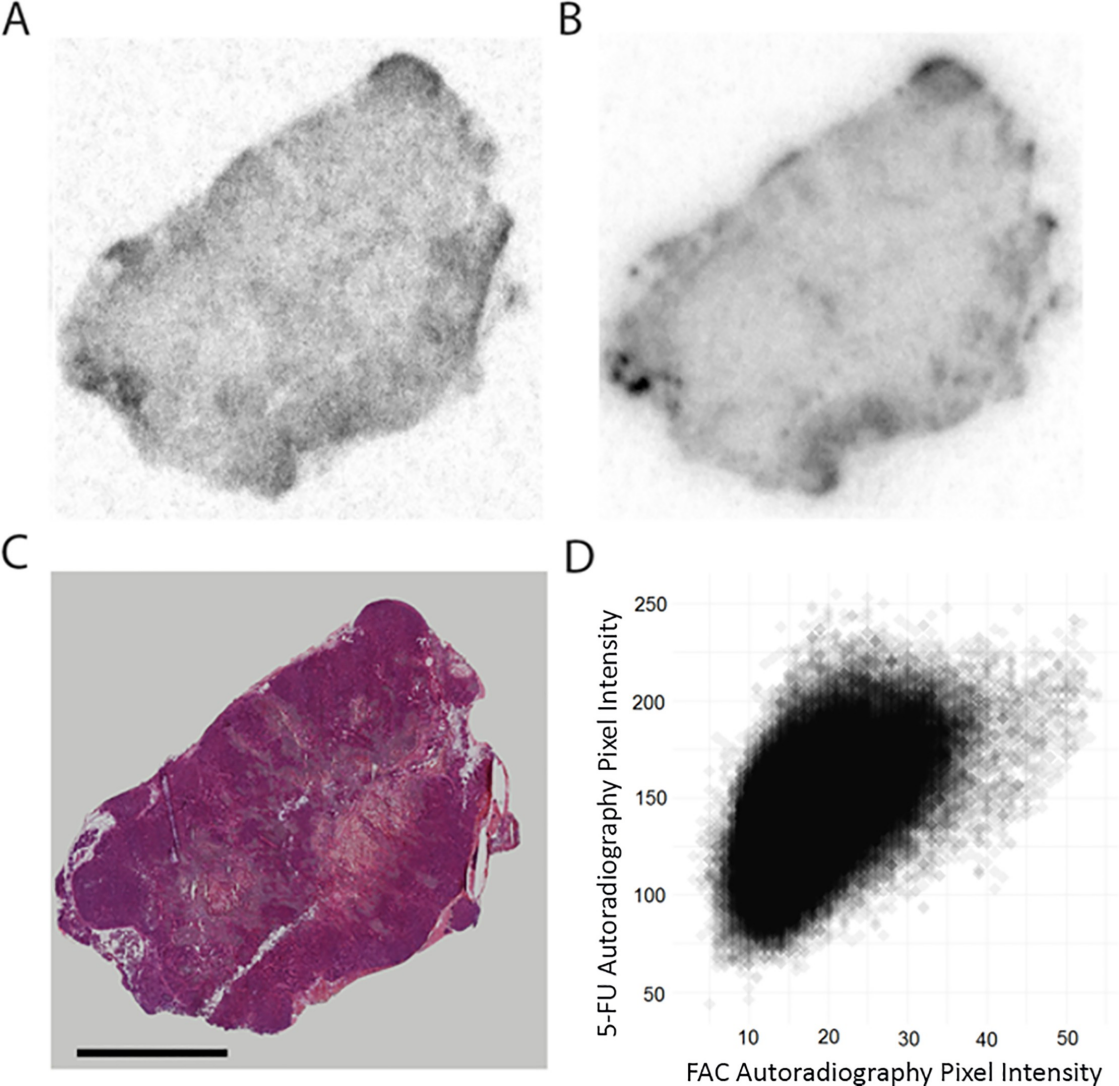
5-FU and FAC intra-tumoral distribution. Autoradiography of [^14^C]-5-FU (A) and of [^18^F]-FAC (B) in an organoid tumor section. H&E staining of that tumor section (C). Scale bar = 5 mm. Scatter plot of the pixel-to-pixel comparison for [^18^F]-FAC and [^14^C]-5-FU autoradiography signal (D).

**Fig 5:**
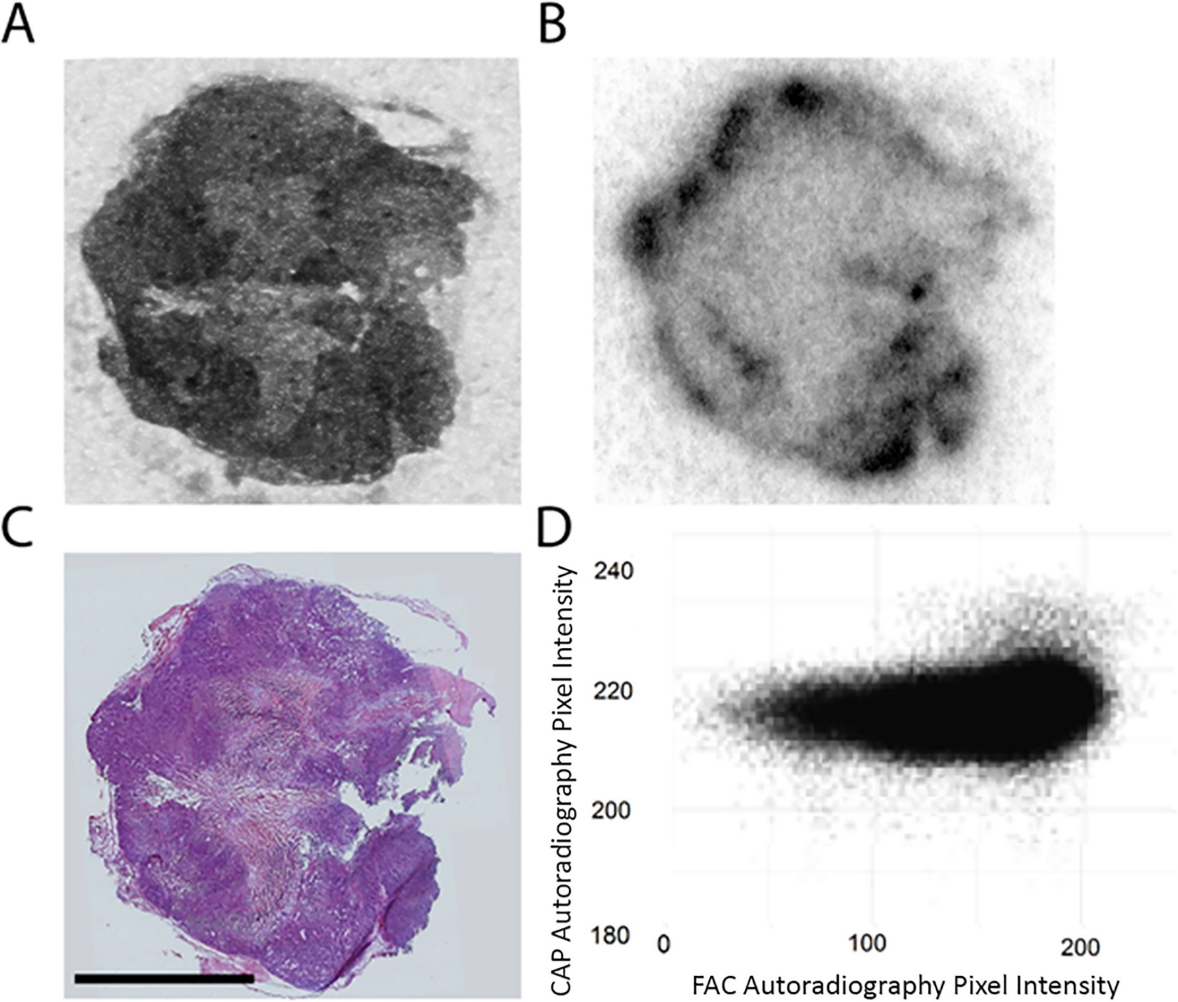
Capecitabine and FAC intra-tumoral distribution. Autoradiography of [^3^H]-capecitabine (A) and of [^18^F]-FAC (B) in an organoid tumor section. H&E staining of that tumor section (C). Scale bar = 5mm. Scatter plot of the pixel-to-pixel comparison for [^18^F]-FAC and [^3^H]-capecitabine autoradiography signal (D).

**Fig 6:**
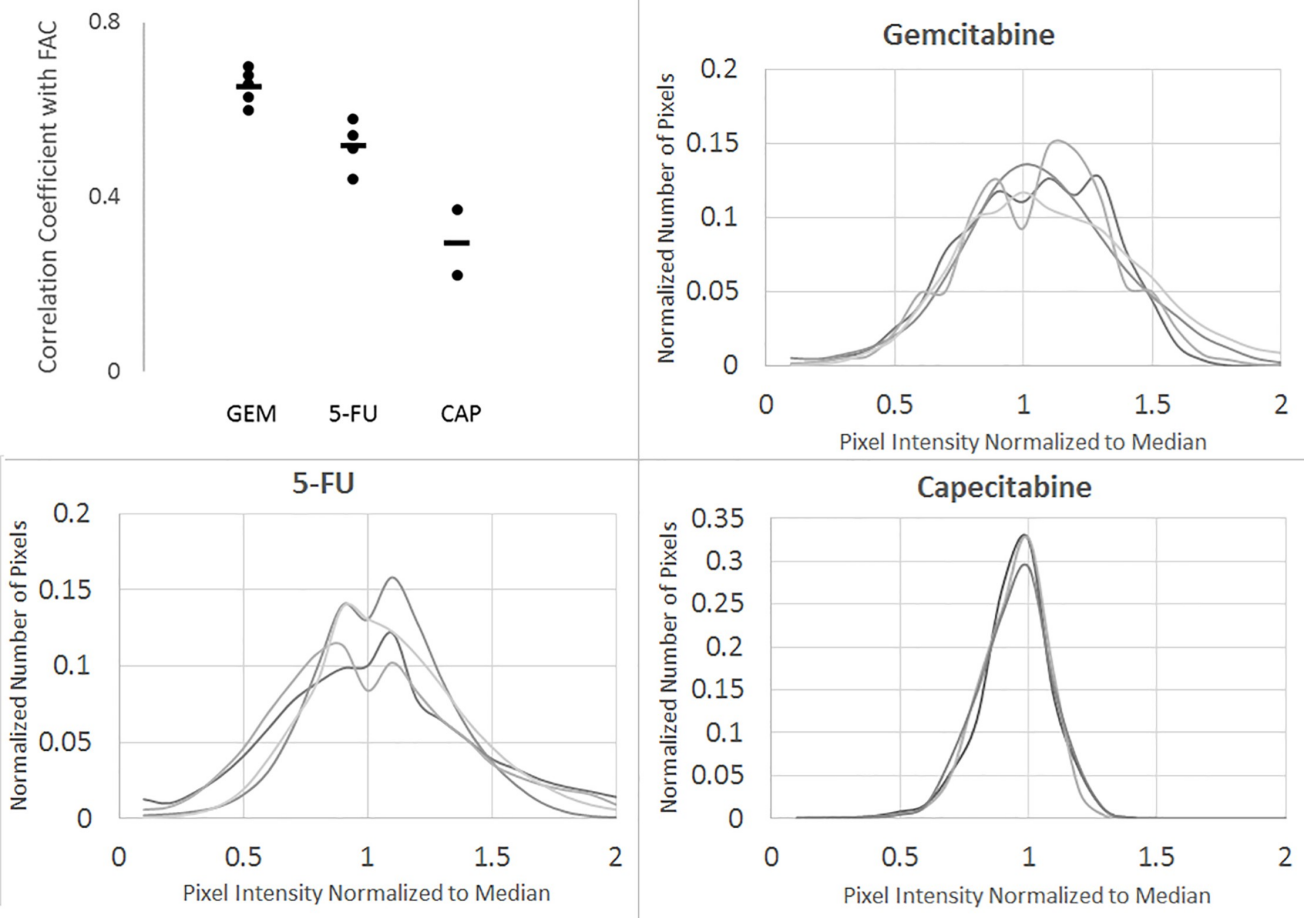
Comparison of gemcitabine, 5-FU and capecitabine drug distribution. (A) Dots show the correlation coefficient of pixels values for FAC/GEM (n = 4), FAC/5-FU (n = 4), and FAC/CAP (n = 3) autoradiography. Lines show the mean value. Histograms of the Gemcitabine (B), 5-FU (C) and Capecitabine (D) activity distribution in the non-necrotic portions of the tumor.

Figs 4 and 5 show the distribution of [^18^F]-FAC compared to [^14^C]-5-FU and [^3^H]-capecitabine, respectively. For reference, a matched H&E image is included. The autoradiographs of sections taken from 4 mice for 5-FU and 3 mice for capecitabine were used to generate the scatter plot of correlated pixel intensities. The complete image sets are shown in S4 and S5 Figs. Again, ^18^F autoradiography shows heterogeneous accumulation of [^18^F]-FAC in the tumor, which is matched by [^14^C]-5-FU. However, ^3^H signal from capecitabine shows a much more uniform distribution across the tumor section. This same pattern was observed in all studied tumors. This results in a weak to nonexistent correlation between [^18^F]-FAC and [^3^H]-capecitabine-derived activity. In contrast, there is a good correlation between [^18^F]-FAC and [^14^C]-5-FU derived activity, that is only slightly less than between [^18^F]-FAC and [^14^C]-gemcitabine. Visually, the hotspots on the [^14^C]-5-FU autoradiographs correspond to the [^18^F]-FAC hotspots. Thus, it seems reasonable that 5-FU, FAC and gemcitabine share similar intra-tumor distribution. The complete sets of correlation coefficients (drug versus [^18^F]-FAC) are plotted in Fig 6A. To illustrate this further, we constructed histograms of the activity distribution in the non-necrotic portions of the tumor (Fig 6B). The distributions of [^14^C]-gemcitabine and [^14^C]-5FU associated activity all display a high degree of skew or are bimodal, representing the fact that every section examined showed very non-homogenous distribution of drug. By contrast the [^3^H]-capecitabine-derived sections all yielded tight normal histograms, consistent with homogenous distribution of activity.

### Drug uptake in hypoxic tissue

**Fig 7:**
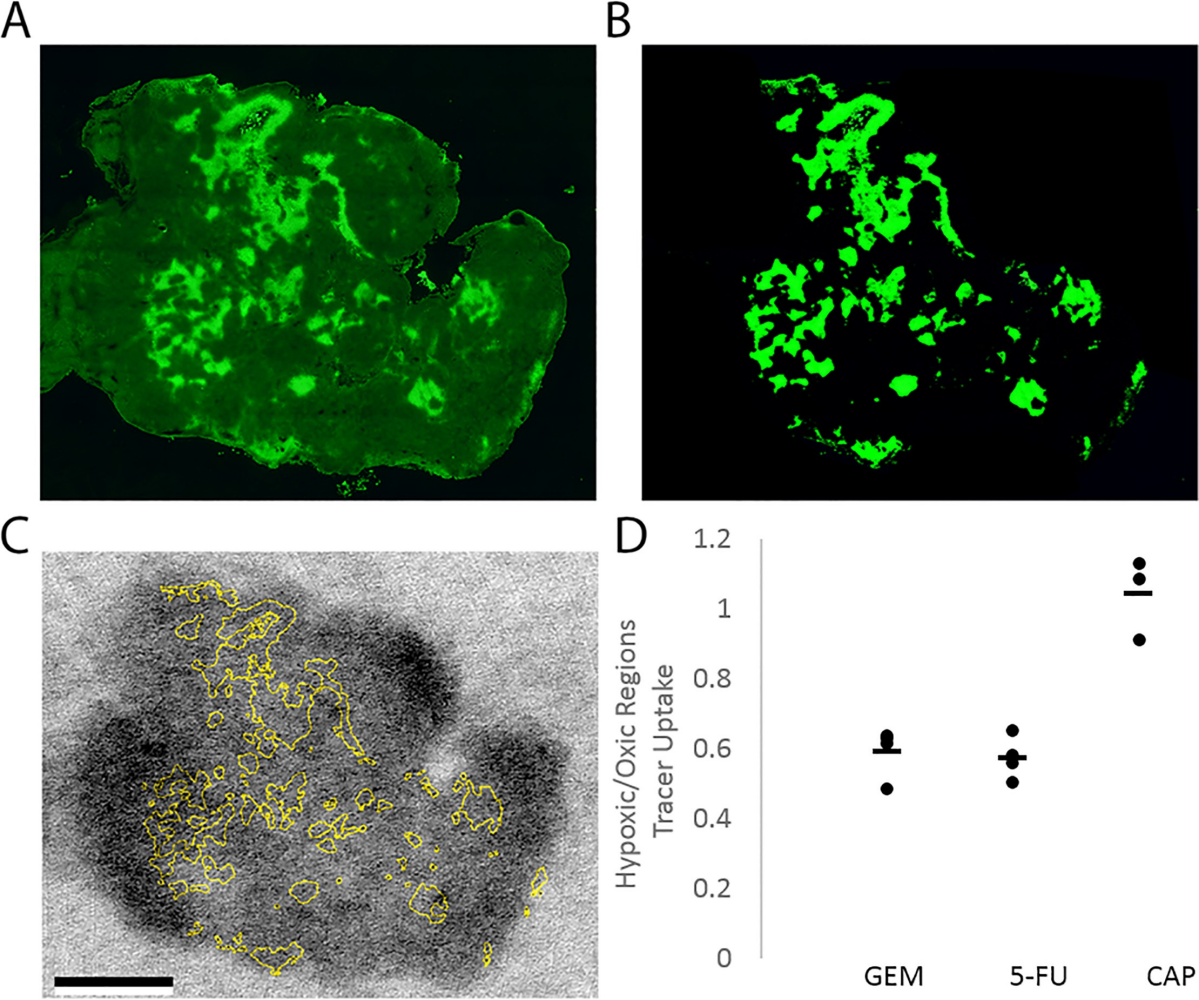
Establishing tracer activity in hypoxic vs oxic regions of the tumor sections. Pimonidazole staining (green) of an organoid tumor section (A). Segmentation of the tumor (green) according to Otsu thresholding (B). The boundaries of the hypoxic regions from (B) are overlaid on top of the ^14^C-gemcitabine autoradiography image (C). The ratio of activity in hypoxic relative to normoxic pixels for gemcitabine, 5-FU and capecitabine (D). Dots represent the value for each mouse (n = 3 for gemcitabine, n = 4 for 5-FU and n = 3 for capecitabine). The line represents the mean value. Scale bar is 5 mm.

As a further indication of drug penetration into the tumor, we analyzed uptake of [^14^C]-gemcitabine, [^14^C]-5-FU and [^3^H] capecitabine in hypoxic versus oxic regions of the tumor. We exposed tumor sections containing one drug or the other to the same autoradiography plate. Tumors were segmented into hypoxic and oxic regions by applying an automated thresholding procedure to pimonidazole images. We measured the hypoxic to oxic drug uptake ratio by measuring the autoradiography signal in hypoxic and in oxic regions, which were delineated on the pimonidazole staining of the section (Fig 7). The lower percentage injected dose per gram for [^14^C]-5-FU in the autoradiographic images of these sections yielded more noisy images compared to that of [^14^C]-gemcitabine; yet the relative hypoxic to oxic ratios were similar between the drugs (0.56 ± 0.07for gemcitabine and 0.58 ± 0.05 for 5-FU). For capecitabine, signal was homogeneous throughout the tumor section.

## Discussion

In this study, we used a PET imaging agent [^18^F]-FAC, that we have shown to be an excellent surrogate imaging agent for gemcitabine, to compare with the tumor microdistribution of other anti-cancer drugs 5-FU and capecitabine. We employed a transplantable tumor model established from an organoid culture of a KPC tumor, which displayed a moderate level of stromal enrichment: collagen bands were present throughout the tumor, as was hyaluronic acid. Most importantly, this PDAC mouse model exhibited the barriers against drug penetration that are considered a possible reason of treatment failure in pancreatic tumors.

We showed that 5-FU is very similarly distributed to gemcitabine, an expected result if the barriers to drug penetration are common to both drugs. The restricted penetration of gemcitabine has been reported in another cancer model [19]. Capecitabine-metabolites had superior penetration i.e. they were homogeneously distributed through the tumor, though the clinical significance of this is not clear, since the radiolabel would be retained on both active and inactive metabolites.

One explanation for the different behavior of 5-FU, gemcitabine and capecitabine is as follows. The desmoplastic microenvironment and high tumor interstitial pressure ensures that small molecules can only penetrate slowly into the tumor mass. Their diffusion away from the blood vessels will be in competition with their uptake and entrapment by tumor cells. For gemcitabine, uptake is through nucleoside transporters [20, 21] followed by phosphorylation by deoxycytidine kinase, which traps the drug within the cell. Capecitabine is metabolized to 5-FU–the final step occurring inside the tumor–and 5-FU is converted to a phosphorylated metabolite in one of two ways: through direct phosphorylation to fluorouridine monophosphate via orotate phosphoribosyl transferase and in a two-step reaction to fluorodeoxyuridine monophosphate. However, 5-FU is also catabolized in a non-toxic pathway via fluoro dihydrouracil to fluoro-β-alanine (FBAL). This complicates interpretation of the autoradiography images, particularly with the assumption that the autoradiography signal represents therapeutic potential. For 5-FU, the ^14^C radiolabel was positioned on C2, which is lost from FBAL and so this species should not contribute to the signal, but the tritium label on capecitabine would be retained in FBAL.

It has been shown clinically with hypoxia radiotracers and hypoxic probes that most pancreatic tumors are hypoxic [22, 23]. Hypoxia is one of the factors that has been proposed as an indicator for pancreatic tumor metastasis and is associated with poor prognosis and the epithelial to mesenchymal transition [24]. The mouse model used in this study generated tumors with large hypoxic regions, from which gemcitabine and 5-FU were largely excluded, highlighting the need for effective options for this refractory population. A hypoxia-activated pro-drug, Evofosfamide recently almost achieved a statistical improvement in outcome in a large phase III trial of pancreatic patients [25], and the patients were not screened for tumor hypoxia. Yet there is ample pre-clinical data to suggest that hypoxic regions of the tumor are not exposed to significant concentrations of currently available cytotoxic, and some form of hypoxia-targeting should be beneficial.

Our next steps will be to perform small animal microPET imaging of tumor bearing animals in order to study and quantify drug uptake and distribution non-invasively.

## Conclusions

Our studies confirm that gemcitabine is unevenly distributed throughout pancreatic tumors and extend this finding to 5-FU. However, this uneven distribution was not imposed by the tumor on small molecules *per se*, as capecitabine metabolites were uniformly distributed through the tumor mass. Whether this represents a therapeutic advantage would depend on the profile of the metabolites, information that is not obtainable from radiolabels alone.

## Supporting information

S1 Fig Catabolism of capecitabine and 5-FU inactive compounds. The blue dot shows the ^3^H location and the red dot, the ^14^C location. The ^14^C atom of 5-FU is lost during catabolism (inactivation) but the ^3^H atom remains on the inactive compounds of capecitabine.
(DOCX)

S2 Fig Enhancing signal-to noise ratio in ImageJ by registering 3–5 adjacent [^14^C]-5-FU autoradiography sections (here annotated A, B, C) and generating a median image (E). The difference in profile (yellow line) between the image of a single tumor section and the median image generated from registering three section is shown in a plot (F).
(DOCX)

S3 Fig Autoradiography of [^14^C]-Gemcitabine (A) and of [^18^F]-FAC (B) in four organoid tumor sections. Pimonidazole and H&E staining of that tumor section (C and D). Scale bar = 5 mm.
(DOCX)

S4 Fig Autoradiography of [^14^C]-5-FU (A) and of [^18^F]-FAC (B) in four organoid tumor sections. Pimonidazole and H&E staining of that tumor section (C and D). Scale bar = 5 mm.
(DOCX)

S5 Fig Autoradiography of [^3^H]-Capecitabine (A) and of [^18^F]-FAC (B) in three organoid tumor sections. Pimonidazole and H&E staining of that tumor section (C and D). One [^18^F]-FAC autoradiography image is missing (middle tumor) due to an injection problem. Scale bar = 5 mm.
(DOCX)
