# Supplementary material for: Comparing the intra-tumoral distribution of Gemcitabine, 5-Fluorouracil, and Capecitabine in a murine model of pancreatic ductal adenocarcinoma
Source: PLoS One. 2020 Apr 16;15(4):e0231745. doi: 10.1371/journal.pone.0231745 (PMC7162455; doi:10.1371/journal.pone.0231745)
Supplement: S1 Fig — The blue dot shows the 3H location and the red dot, the 14C location. The 14C atom of 5-FU is lost during catabolism (inactivation) but the 3H atom remains on the inactive compounds of capecitabine. (DOCX) [file pone.0231745.s001.docx]

**
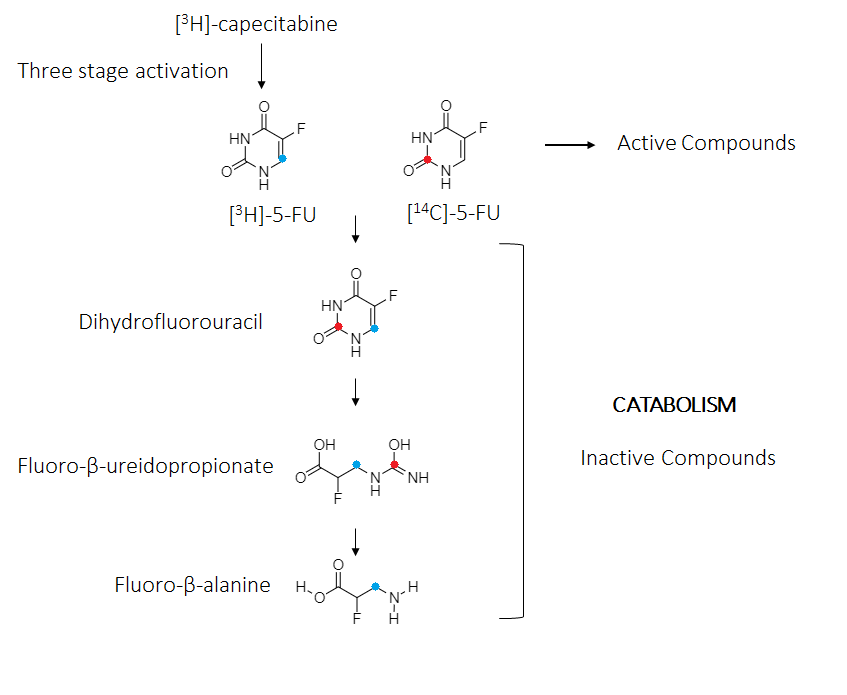
**

**Fig. S1: Catabolism of capecitabine and 5-FU inactive compounds**. The blue dot shows the ^3^H location and the red dot, the ^14^C location. The ^14^C atom of 5-FU is lost during catabolism (inactivation) but the ^3^H atom remains on the inactive compounds of capecitabine.
