# Supplementary material for: Comparing the intra-tumoral distribution of Gemcitabine, 5-Fluorouracil, and Capecitabine in a murine model of pancreatic ductal adenocarcinoma
Source: PLoS One. 2020 Apr 16;15(4):e0231745. doi: 10.1371/journal.pone.0231745 (PMC7162455; doi:10.1371/journal.pone.0231745)
Supplement: S2 Fig — Enhancing signal-to noise ratio in ImageJ by registering 3–5 adjacent [14C]-5-FU autoradiography sections (here annotated A, B, C) and generating a median image (E). The difference in profile (yellow line) between the image of a single tumor section and the median image generated from registering three section is shown in a plot (F). (DOCX) [file pone.0231745.s002.docx]

**
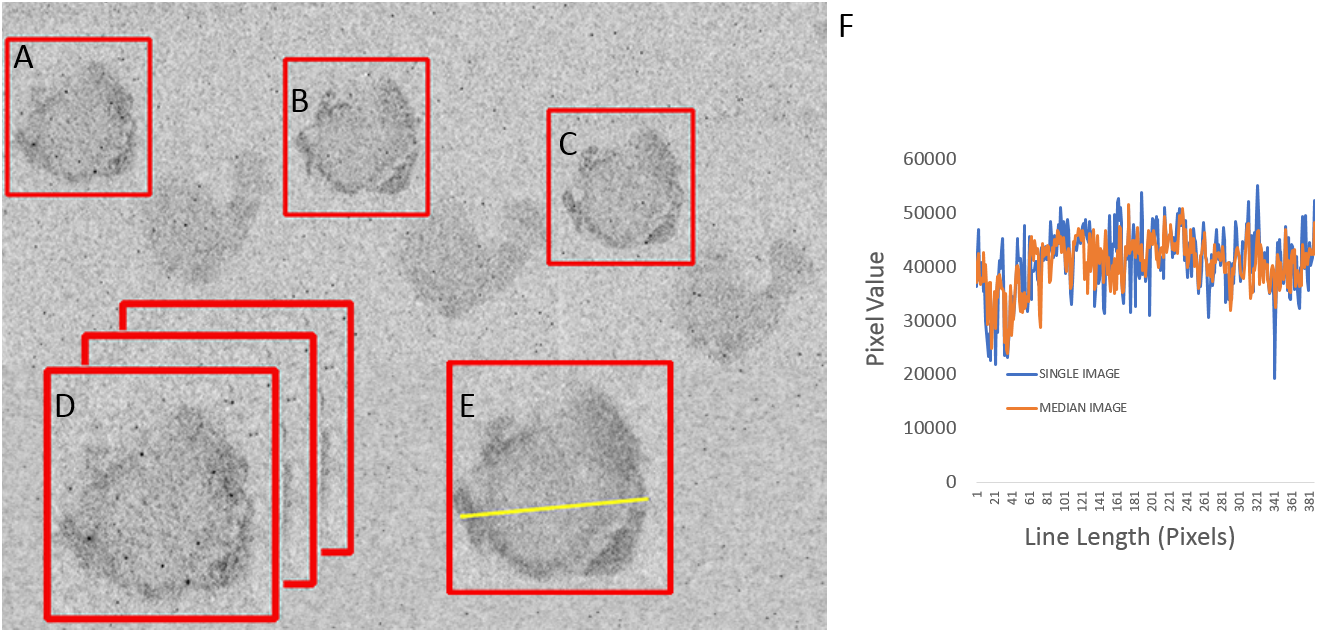
**

**Fig. S2:** Enhancing signal-to noise ratio in ImageJ by registering 3-5 adjacent [^14^C]-5-FU autoradiography sections (here annotated A, B, C) and generating a median image (E). The difference in profile (yellow line) between the image of a single tumor section and the median image generated from registering three sections is shown in a plot (F).
