# Supplementary material for: Comparing the intra-tumoral distribution of Gemcitabine, 5-Fluorouracil, and Capecitabine in a murine model of pancreatic ductal adenocarcinoma
Source: PLoS One. 2020 Apr 16;15(4):e0231745. doi: 10.1371/journal.pone.0231745 (PMC7162455; doi:10.1371/journal.pone.0231745)
Supplement: S3 Fig — Autoradiography of [14C]-Gemcitabine (A) and of [18F]-FAC (B) in four organoid tumor sections. Pimonidazole and H&E staining of that tumor section (C and D). Scale bar = 5 mm. (DOCX) [file pone.0231745.s003.docx]

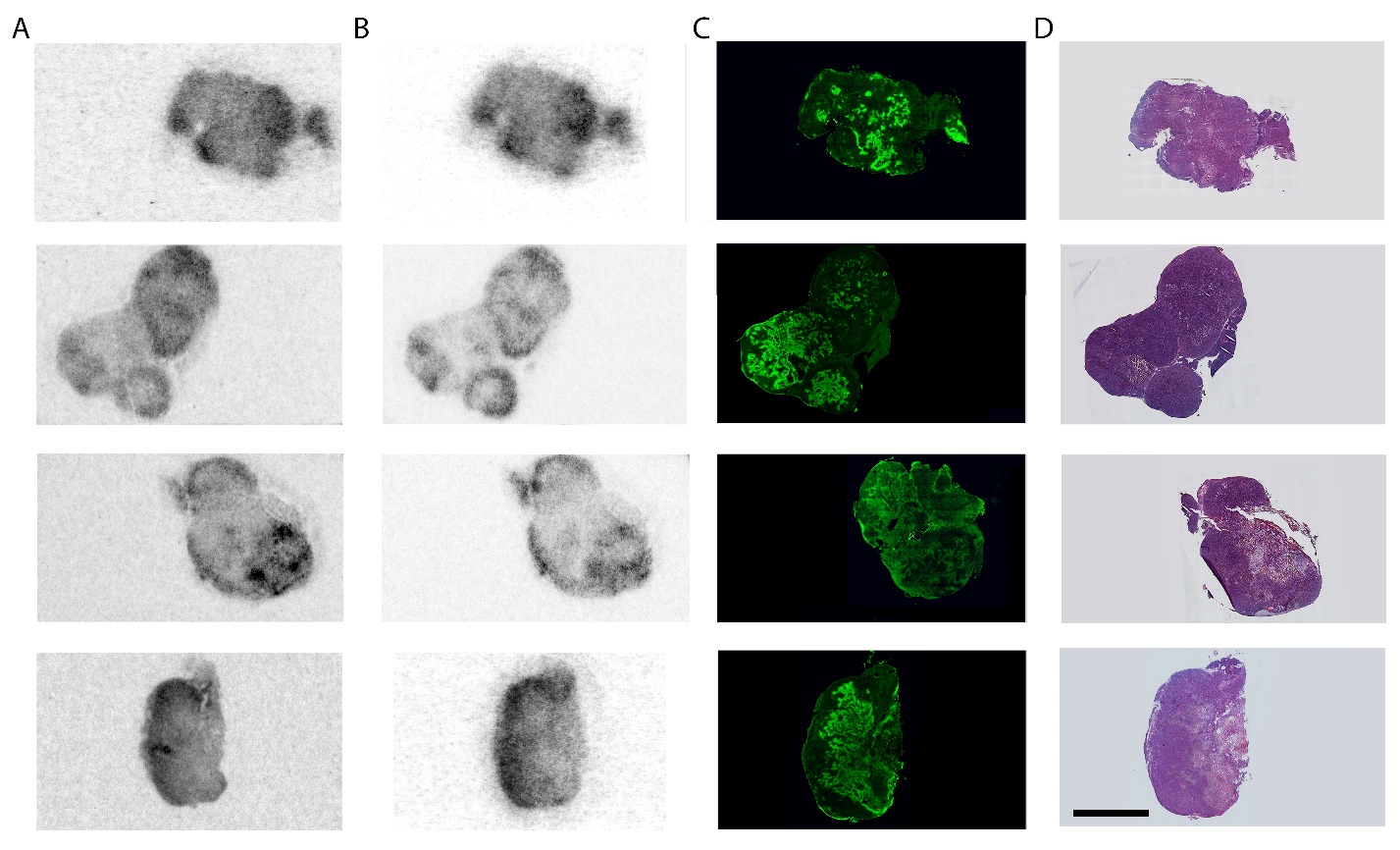


**Fig. S3:** Autoradiography of [^14^C]-Gemcitabine (A) and of [^18^F]-FAC (B) in four organoid tumor sections. Pimonidazole and H&E staining of that tumor section (C and D). Scale bar = 5 mm.
