# Supplementary material for: Comparing the intra-tumoral distribution of Gemcitabine, 5-Fluorouracil, and Capecitabine in a murine model of pancreatic ductal adenocarcinoma
Source: PLoS One. 2020 Apr 16;15(4):e0231745. doi: 10.1371/journal.pone.0231745 (PMC7162455; doi:10.1371/journal.pone.0231745)
Supplement: S5 Fig — Autoradiography of [3H]-Capecitabine (A) and of [18F]-FAC (B) in three organoid tumor sections. Pimonidazole and H&E staining of that tumor section (C and D). One [18F]-FAC autoradiography image is missing (middle tumor) due to an injection problem. Scale bar = 5 mm. (DOCX) [file pone.0231745.s005.docx]

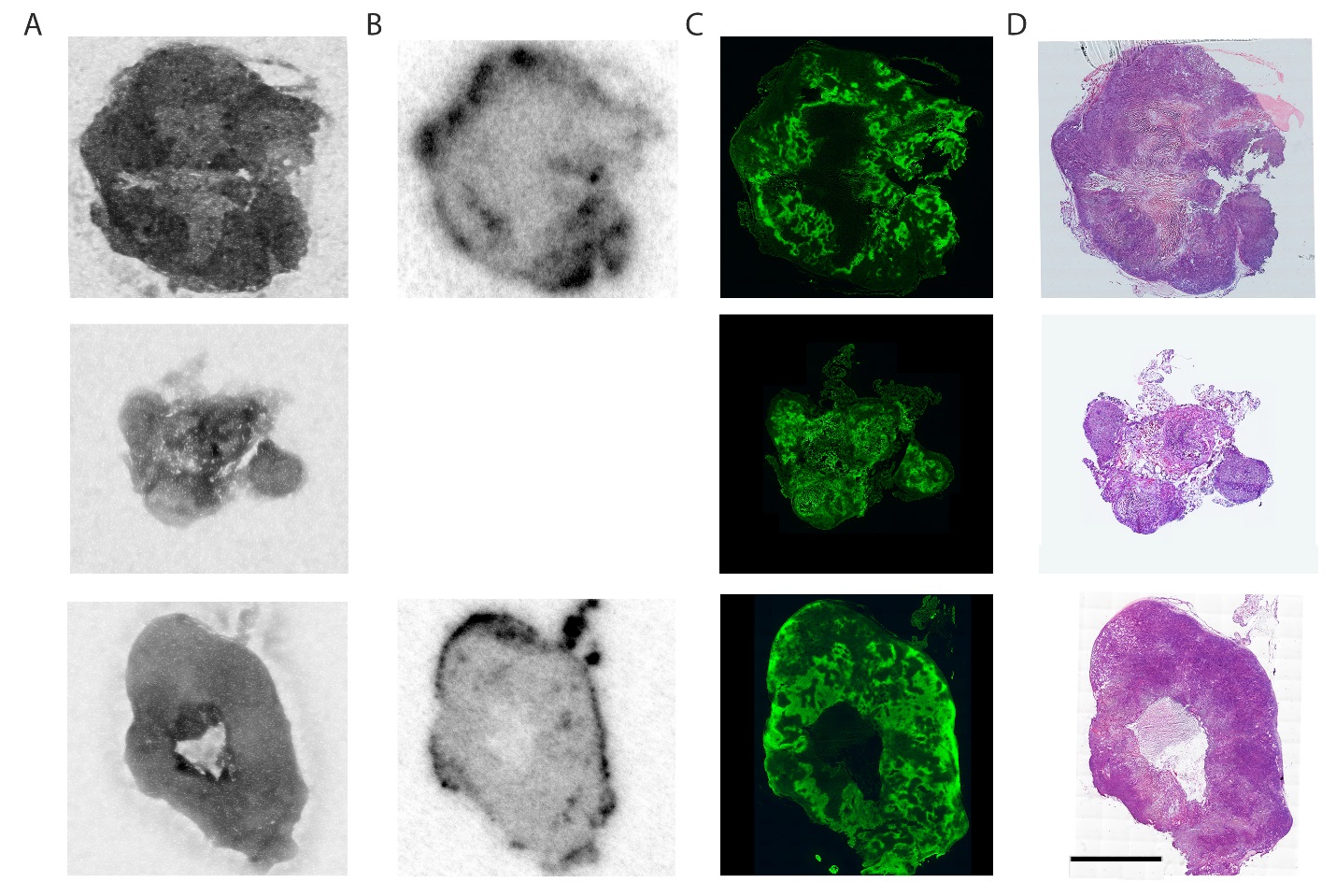


**Fig. S5:** Autoradiography of [^3^H]-capecitabine (A) and of [^18^F]-FAC (B) in three organoid tumor sections. Pimonidazole and H&E staining of that tumor section (C and D). One [^18^F]-FAC autoradiography image is missing (middle tumor) due to an injection problem. Scale bar = 5 mm.
